# Island formation of Er(trensal) single-ion magnets on graphene observed on the micrometer scale†

**DOI:** 10.1039/d1ra00783a

**Published:** 2021-03-02

**Authors:** Jan Dreiser, Christian Wäckerlin, Michele Buzzi, Kasper S. Pedersen, Jesper Bendix

**Affiliations:** Swiss Light Source, Paul Scherrer Institut CH-5232 Villigen PSI Switzerland jan.dreiser@psi.ch; Institute of Physics (IPHYS), Ecole Polytechnique Fédérale de Lausanne (EPFL) CH-1015 Lausanne Switzerland; Surface Science and Coating Technologies, Empa, Swiss Federal Laboratories for Materials Science and Technology Überlandstrasse 129, 8600 Dübendorf Switzerland; Department of Chemistry, Copenhagen University DK-2100 Copenhagen Denmark; Department of Chemistry, Technical University of Denmark DK-2800 Kgs. Lyngby Denmark

## Abstract

We have studied the morphology of Er(trensal) single-ion molecular magnets adsorbed on graphene/Ru(0001) using X-ray photoemission electron microscopy (X-PEEM). By exploiting the elemental contrast at the erbium M_5_ edge we observe the formation of molecular islands of homogeneous height with a lateral size of several micrometers. The graphene/Ru(0001) substrate exhibits two different signal levels in bright-field low-energy electron microscopy (LEEM) and in X-PEEM, which are ascribed to the presence of small-angle rotational domains of the graphene lattice. We find that the Er(trensal) molecules form islands solely on the bright areas, while the remaining dark areas are empty. Our results are important for the growth and study of the molecule–inorganic hybrid approach in spintronics schemes.

## Abstract 2

The island formation of Er(trensal) single-ion molecular magnets on graphene/Ru(0001) has been studied using X-ray photoelectron emission microscopy.

## Graphical abstract

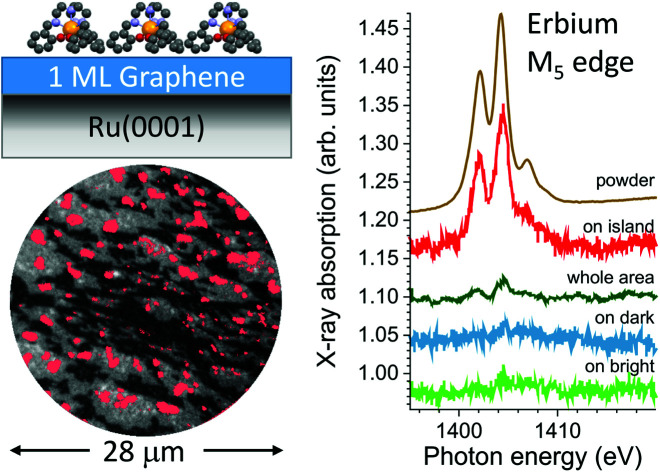

## 1. Introduction

The investigation of the growth properties of molecular nanomagnets on flat surfaces^1–3^ is highly relevant for molecular spintronics schemes involving heterostacks of different materials such as metals, two-dimensional (2D) materials like graphene,^4–6^ hexagonal boron nitride^7,8^ and other layered materials that are currently receiving enormous attention.^9,10^ Here, the interface between different constituent layers^11,12^ plays a crucial role in creating new properties arising from the contact of two different materials. Single-ion molecular magnets (SIMs), which exhibit slow relaxation of their magnetization,^13–17^ are interesting candidates to be used as modular building blocks to produce, *e.g.*, addressable arrays of magnetic bits,^18^ magnetic insulating layers in tunnel junctions or to modify the properties of the underlying layers through hybridization of molecular and substrate orbitals.^12^

On graphene, like in many other 2D materials, adsorption is weak and dominated by van der Waals interactions. In turn, this leads to a comparably small modification of the adsorbates' molecular structure and magnetic properties compared to the bulk. Furthermore, it facilitates the formation of ordered arrays or molecular islands through diffusion, which is not possible when adsorbates are strongly attached by covalent bonds.

Here we employ synchrotron based X-ray photoemission electron microscopy (X-PEEM) in order to investigate the organization of Er(trensal) SIMs^19–21^ on graphene (G) grown by chemical vapor deposition on Ru(0001).^22–27^ This technique allows high spatial resolution on the scale of tens of nanometers combined with element specificity. It has been used to study the growth and organization of organic molecules on surfaces,^28–31^ yet, studies involving molecular nanomagnets are very rare.^32^

**Fig. 1:**
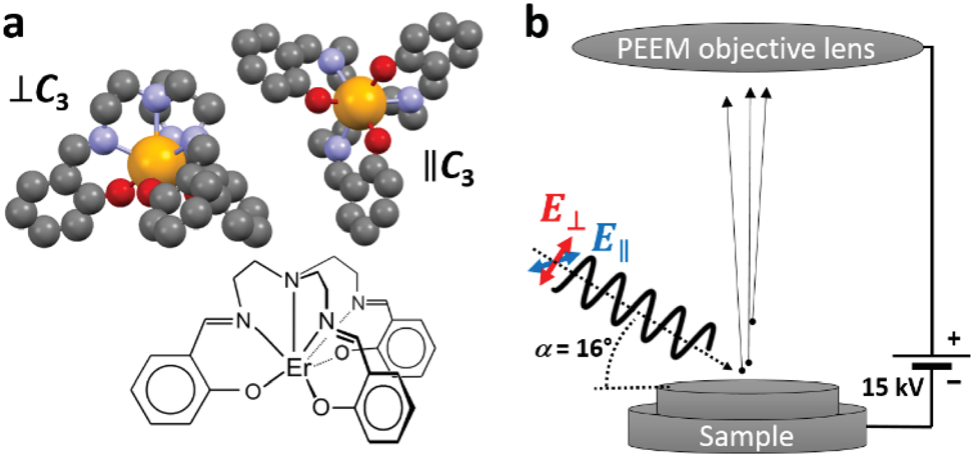
(a) Views of the Er(trensal) molecule along and perpendicular to the molecular *C*_3_ axis and structural scheme. Color code: orange: erbium; red: oxygen; blue: nitrogen; grey: carbon. Hydrogen atoms have been omitted for clarity. The lower scheme illustrates the coordination environment of the Er^3+^ ion and the structure of the ligand. (b) Sketch of the X-PEEM experiment. Electrons are ejected upon illumination with X-rays incident at a grazing angle of *α* = 16°.

The non-planar trigonal pyramidal Er(trensal) molecule is visualized in Fig. 1a. The seven-fold coordinated Er^3+^ ion is subject to a trigonal ligand-field, with the *C*_3_ symmetry axis passing through the apical nitrogen atom. The adsorption conformations of the Er(trensal) molecules strongly depend on the nature of the substrates. Their adsorption and magnetic properties as monolayers have been studied recently by some of us using spatially averaging X-ray magnetic circular dichroism (XMCD) and scanning tunneling microscopy (STM) at low temperatures of a few Kelvin. Er(trensal) exhibits several energetically equivalent adsorption conformations on the Au(111) surface.^33^ This is also the case on Ni/Cu(001)^33^ and Ru(0001) surfaces.^34^ In contrast, on G/Ru(0001) and G/Ir(111) the molecules self assemble into ordered islands, reminiscent of the organization of the molecules in the molecular single crystal. The orientation of all molecular *C*_3_ axes perpendicular to the surface plane gives rise to a net magnetic anisotropy of the molecular ensemble, which was observed by angle dependent XMCD measurements.^34^ A magnetic hysteresis was not observed, while it was also not expected in view of the magnetic relaxation timescales of Er(trensal) compared to the field sweeping speed in the XMCD magnetic field scan. It was difficult to obtain images on a larger scale in the STM study on G/Ru(0001), because of the very weak adsorption of the molecules with the tendency toward tip instabilities.

The XMCD results in ref. 34 were obtained using a relatively large X-ray spot with a size of ∼0.5 mm^2^. In the present study we complement the previous results by probing at a lateral scale to several tens of micrometers, which allows to obtain a mesoscopic view on this molecule/graphene/inorganic hybrid system. In the X-PEEM, the sample is illuminated with monochromatic X-ray photons of tunable energy and variable polarization. The ejected secondary electrons, resulting from the decay of the photoexcited states, are collected through an objective lens as shown in the sketch in Fig. 1b. After passing through the electron microscope optics, they are intensified by a microchannel plate (MCP), converted to visible light using a fluorescent screen and detected by a charge coupled device (CCD) camera, yielding a lateral resolution down to 50 nm. The technique is very surface sensitive because of the shallow electron escape depth of a few nanometers. We have also analyzed bright-field low-energy electron microscopy (LEEM) and micro-spot low-energy electron diffraction (μ-LEED) images, where the back-reflected and diffracted electrons are imaged, respectively.

## 2. Methods

### 2.1 Sample preparation

Er(trensal), where H_3_trensal = 2,2′,2′′-tris(salicylideneimino) triethylamine, was synthesized according to literature procedures.^19,20^ The Ru(0001) single crystal surface was prepared by repeated sputter-annealing cycles using Ar^+^ ions at an energy of 2 keV and temperatures of up to 1500 K, respectively. Graphene was grown by exposing the hot Ru crystal to a partial pressure of *p* = 10^−6^ mbar of ethylene (C_2_H_2_) for 100 seconds corresponding to an exposure to 75 Langmuirs. The base pressure in the preparation chamber was *p*_0_ = 10^−10^ mbar. After preparation, the sample was transferred to the X-PEEM chamber without breaking the vacuum. The Er(trensal) molecules were deposited from a Knudsen cell held at a temperature of 560 K mounted directly at the X-PEEM ultra-high vacuum chamber onto the prepared substrate at room temperature.

The Er(trensal) polycrystalline powder reference sample was prepared by pressing the polycrystalline powder of molecules into indium foil, which was adhered to the sample holder.

### 2.2 Low-energy electron microscopy, micro low energy electron diffraction and X-ray photoelectron emission microscopy

All measurements were performed at room temperature using the X-PEEM instrument (Elmitec) at the SIM beam line of the Swiss Light Source, Paul Scherrer Institut.^35,36^ In all measurements the sample was biased at a potential of *U*_bias_ = 15 kV with respect to the objective lens. For the low-energy electron microscopy (LEEM) and micro low-energy electron diffraction (μ-LEED) measurements, the sample was exposed to an electron beam from an internal source with an energy of up to *E*_e_ = 50 eV relative to the sample potential.

For the X-PEEM measurements, the SIM beam line was employed as a monochromatic, linearly polarized photon source. Spatially resolved X-ray absorption spectra were recorded by repeatedly taking X-PEEM images while incrementing the photon energy by a small value. Static images were acquired at the C K edge (*E* = 285.7 eV) and at the Er M_5_ edge (*E* = 1404.4 eV). In each case reference images were taken at the pre-edge with a slightly lower photon energy (*E* = 283.7 eV and *E* = 1397.7 eV, respectively). The static images were corrected for a small lateral drift and divided by the respective pre-edge images. Raw images are shown in the Fig. S1 and S2 of the ESI.† All image operations were performed using the ImageJ software. The LEEM image was normalized to a defocused (flat field) image in order to account for spatially inhomogeneous sensitivity of the used multi-channel plate.

### 2.3 X-ray absorption spectroscopy

The X-ray absorption measurement on the powder sample was performed at the X-Treme^37^ beam line of the Swiss Light Source, using a defocused X-ray spot (∼0.5 mm^2^).

## 3. Results and discussion

In the following the LEEM and μ-LEED results recorded on the bare G/Ru(0001) substrate will be discussed. Later on, the X-PEEM results obtained on Er(trensal)/G/Ru(0001) will be analyzed.

### 3.1 LEEM and μ-LEED

**Fig. 2:**
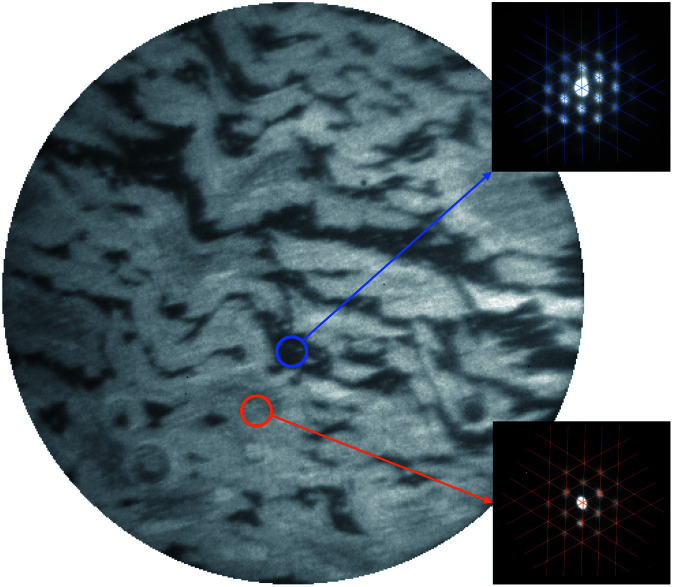
(Main panel) LEEM image recorded on G/Ru(0001) at an incident electron energy of 11 eV. The field of view is 28 μm. (Insets) μ-LEED patterns recorded at an electron energy of 30 eV at the two different spots (∼2 μm diameter) marked by the circles. The lines are guides to the eyes.

In Fig. 2, a bright-field LEEM image of the G/Ru(0001) substrate recorded at an incident electron energy of 11 eV is shown. Two shades of gray corresponding to two signal levels are observed which are attributed to the presence of rotational domains having different angles between the graphene and the underlying Ru(0001) lattice.^25,27^ The two different signal levels arise from different electron reflectivities as observed in G/Ir(111)^38^ and in G/Pd(111)^39^ which are related to small work function differences.^39^ The equal presence of graphene in both the dark and the bright domains is evidenced by the μ-LEED measurements (*E*_el_ = 30 eV) shown in the insets of Fig. 2. The signal collected locally (spot size of ∼2 μm diameter), as indicated by the small circles in the main panel, exhibits in both cases the satellite peaks centered around the (0,0) reflection of the Ru(0001) substrate. This pattern indicates the presence of the well-known moiré pattern arising from the mismatch between the graphene and Ru(0001) lattices.^22,24,27^ According to literature reports, the difference in rotation angle in G/Ru(0001) is less than Δ*ϕ* = ∼1°.^25,27^ The angle uncertainty of the μ-LEED measurements in the present work is estimated to be Δ*ϕ* = 2° through the line patterns plotted in the insets of Fig. 2, which are not rotated with respect to each other.

### 3.2 X-PEEM

**Fig. 3:**
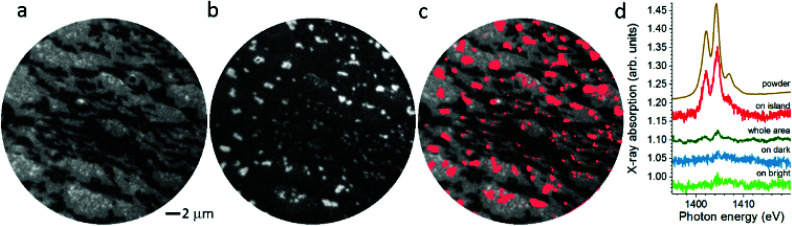
X-PEEM images recorded at room temperature on *in situ* prepared Er(trensal)/G/Ru(0001). (a) Linear dichroism image recorded at the carbon K edge. (b) Image taken at the erbium M_5_ absorption edge. The imaging area is the same as in (a). (c) Overlay of Er(trensal) islands observed in panel (b) shown in red and the carbon XLD image in panel (a). (d) X-ray absorption spectra at the erbium M_5_ edge obtained on different positions as marked in the plot and on a powder sample of Er(trensal). The powder spectrum has been rescaled to match with the island spectrum.

In Fig. 3, X-PEEM images taken on Er(trensal)/G/Ru(0001) at different photon energies along with X-ray absorption spectra recorded in the vicinity of the C K edge and the Er M_5_ absorption edge are shown. In Fig. 3a a C K edge X-ray linear dichroism image, obtained according to1
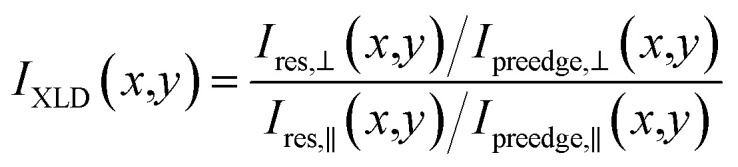
is presented. Here, *x*,*y* refer to the coordinate axes in the imaging plane *I*_res_ and *I*_preedge_ are the images taken at the absorption edge and at the pre-edge photon energies as specified in the Experimental Section. The symbols ⊥ and ∥, respectively, denote the orientation of the oscillating electric field *E*_osc_ of the linearly polarized X-ray beam with respect to the sample plane. Note that due to the grazing angle of *α* = 16°, *E*_osc,⊥_ is only quasi-perpendicular to the surface plane, while *E*_osc,∥_ lies perfectly in that plane.

In the XLD image, again two signal levels are observed similar to the LEEM image on G/Ru(0001) shown in Fig. 2. A few brighter speckles occur, too. In analogy to the previous analysis of the LEEM image, we attribute the contrast to the presence of different rotational domains, which have been shown to possess different work function in the case of G/Pd(111).^39^ Note that the contrast is not due to the presence of multilayer graphene, as can be concluded from the analysis of the C K edge spectra taken with the two linear polarizations (not shown here). Also the raw images taken at the pre-edges exhibit this contrast as observed in Fig. S1a, d and S2a, e in the ESI.† This confirms the work function difference, which is connected with the rotational domains.^39^

The image shown in Fig. 3b has been obtained at the Er M_5_ edge. While in the background a weak contrast from the rotational domains is still seen, very intense speckles with uniform intensity appear. In the superposition shown in Fig. 3c it becomes visible that the intense speckles are almost exclusively formed on the brighter domains of G/Ru(0001). The X-ray absorption spectra (*cf*. Fig. 3d), obtained locally at different positions, in comparison with the powder reference data clearly confirm the presence of erbium in the islands. Hence we identify the intense speckles as islands of Er(trensal) molecules. In Fig. 3c, very few speckles do not match with the Er(trensal) islands, that is, they do not contain Er. They might originate from a small amount of contaminants, which were adsorbed during the different steps of the sample preparation. The averaged spectrum denoted by ‘whole area’ reveals a ratio of the main XAS peak *versus* the background of 1.8% ± 0.2%. On the molecular island, a peak-to-background ratio of 15 ± 0.5% is observed. This indicates that the mean coverage is on the order of 12 ± 2% of a full layer of molecules, which is perfectly in line with the particle area analysis yielding a coverage of 12 ± 3%. These numbers are in good agreement with the ones obtained in the previous combined STM-XMCD study.^34^ Furthermore, the spectra shown in Fig. 3d indicate that no traces of Er(trensal) molecules are detected on the seemingly empty dark and bright areas. This suggests that virtually all molecules are engaged in the island formation, and there are no molecules, which are adsorbed on random, *e.g.*, defect sites, in which case a homogeneous but dilute ensemble would be expected. Also, the thermal energy at room temperature is not sufficient to ‘melt’ the molecular islands.

The uniformity of the island appearance in Fig. 3b and the analysis of the peak-to-background ratios show that the islands are composed of a single layer of densely packed Er(trensal) molecules with the surface unit cell described in ref. 34. Furthermore, the exclusive formation of molecular islands on the bright areas can be rationalized by a higher mobility of the molecules on the dark areas, implying the presence of lower diffusion barriers on the dark areas. Another reason could be a larger adsorption energy on the bright areas originating from the slightly different electronic structures of the rotational domains of G/Ru(0001).

## 4. Conclusions

In conclusion graphene grown on Ru(0001) by chemical vapor deposition was characterized by LEEM and by μ-LEED, revealing the full coverage of the Ru(0001) surface by a single layer of graphene. Two different types of domains yielding a different contrast in the LEEM images are found. Our study of Er(trensal) single-ion molecular magnets adsorbed on graphene/Ru(0001) using X-PEEM shows that the molecules form extended 2D islands of up to several micrometers in size, localizing on only one type of the graphene/Ru(0001) domains. The present results are important for the understanding of the growth of non-planar molecular magnets and for potential molecular spintronics applications.

## Conflicts of interest

There are no conflicts to declare.

## Supplementary Material

RA-011-D1RA00783A-s001
