# Supplementary material for: Island formation of Er(trensal) single-ion magnets on graphene observed on the micrometer scale
Source: RSC Adv. 2021 Mar 2;11(16):9421–5. doi: 10.1039/d1ra00783a (PMC8695229; doi:10.1039/d1ra00783a)
Supplement: RA-011-D1RA00783A-s001 [file RA-011-D1RA00783A-s001.pdf]

Electronic Supplementary Material (ESI) for RSC Advances.
This journal is © The Royal Society of Chemistry 2021

Electronic Supplementary Information

Island formation of Er(trensal) single-ion magnets on graphene observed on the micrometer scale

Jan Dreiser,*[ab] Christian Wäckerlin,[bc] Michele Buzzi,[a] Kasper S. Pedersen[de] and Jesper Bendix[d]

[a] Swiss Light Source, Paul Scherrer Institut, CH-5232 Villigen PSI, Switzerland.
[b] Institute of Physics (IPHYS), Ecole Polytechnique Fédérale de Lausanne (EPFL), CH-1015 Lausanne, Switzerland.
[c] Surface Science and Coating Technologies, Empa, Swiss Federal Laboratories for Materials Science and Technology, Überlandstrasse 129, 8600 Dübendorf, Switzerland
[d] Department of Chemistry, Copenhagen University, DK-2100 Copenhagen, Denmark.
[e] Department of Chemistry, Technical University of Denmark, DK-2800 Kgs. Lyngby, Denmark

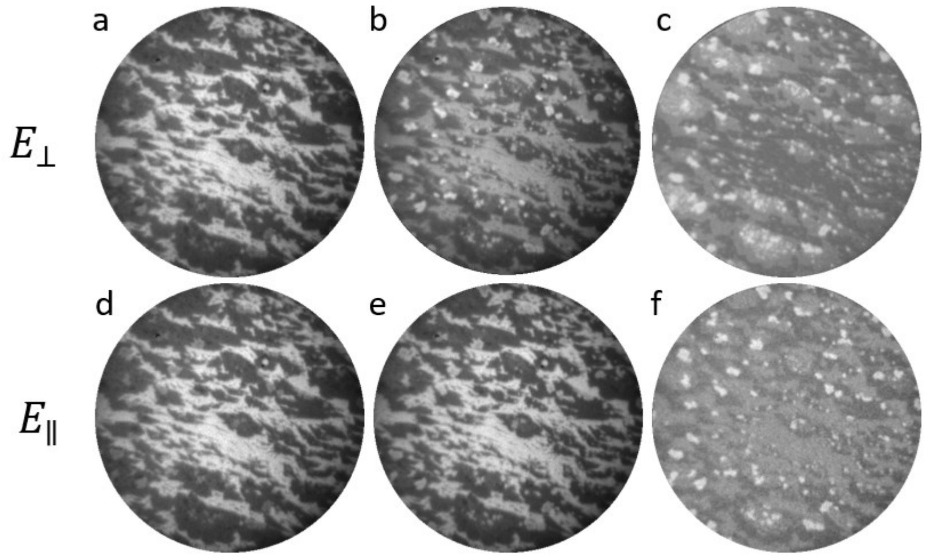

Figure S1. Room-temperature X-PEEM images (28 μm field of view) recorded at different photon energies on Er(trensal)/G/Ru(0001) in the vicinity of the C K edge with variable linear polarization. Images (a-c) and (d-f) were obtained with the oscillating electric field of the X-rays quasi-perpendicular and parallel to the sample surface, respectively. The photon energies were (a,d) 283.7 eV (pre-edge), (b,e) 285.7 eV (on resonance). Images (c) and (f) are the corresponding normalized images obtained by dividing on-resonance one by the pre-edge image.

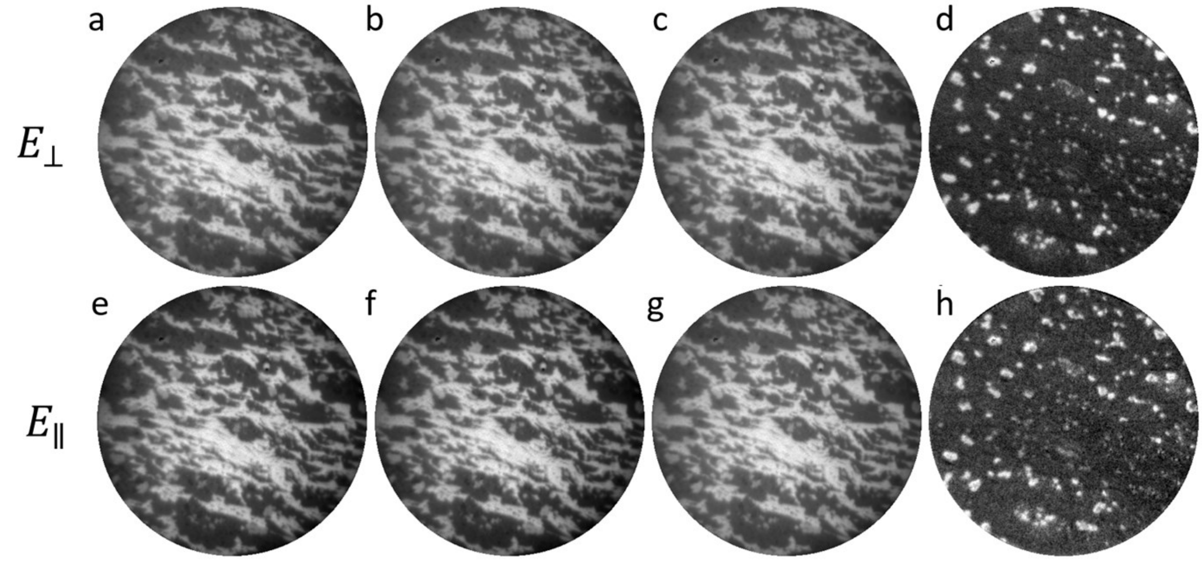

Figure S2. Room-temperature X-PEEM images (28 μm field of view) recorded on Er(trensal)/G/Ru(0001) in the vicinity of the Er M_5 edge with variable linear polarization. Images (a-c) and (e-g) were taken with the oscillating electric field of the X-rays quasi-perpendicular and parallel to the sample surface, respectively. The photon energies were (a,e) 1397.7 eV, (b,f) 1402.0 eV and (c,g) 1404.0 eV. Images (d) and (h) are normalized images obtained by dividing (c) by (a) and (g) by (e), respectively.
